# Nervous System and Intracranial Tumour Incidence by Ethnicity in England, 2001–2007: A Descriptive Epidemiological Study

**DOI:** 10.1371/journal.pone.0154347

**Published:** 2016-05-02

**Authors:** Edward J. Maile, Isobel Barnes, Alexander E. Finlayson, Shameq Sayeed, Raghib Ali

**Affiliations:** 1 Cancer Epidemiology Unit, Nuffield Department of Population Health, Medical Sciences Division, University of Oxford, Oxford, Oxfordshire, United Kingdom; 2 Faculty of Medicine and Health Sciences, Institute of Public Health, United Arab Emirates University, Abu Dhabi, UAE; National Health Research Institutes, TAIWAN

## Abstract

**Background:**

There is substantial variation in nervous system and intracranial tumour incidence worldwide. UK incidence data have limited utility because they group these diverse tumours together and do not provide data for individual ethnic groups within Blacks and South Asians. Our objective was to determine the incidence of individual tumour types for seven individual ethnic groups.

**Methods:**

We used data from the National Cancer Intelligence Network on tumour site, age, sex and deprivation to identify 42,207 tumour cases. Self-reported ethnicity was obtained from the Hospital Episode Statistics database. We used mid-year population estimates from the Office for National Statistics. We analysed tumours by site using Poisson regression to estimate incidence rate ratios comparing non-White ethnicities to Whites after adjustment for sex, age and deprivation.

**Results:**

Our study showed differences in tumour incidence by ethnicity for gliomas, meningiomas, pituitary tumours and cranial and paraspinal nerve tumours. Relative to Whites; South Asians, Blacks and Chinese have a lower incidence of gliomas (p<0.01), with respective incidence rate ratios of 0.68 (confidence interval: 0.60–0.77), 0.62 (0.52–0.73) and 0.58 (0.41–0.83). Blacks have a higher incidence of meningioma (p<0.01) with an incidence rate ratio of 1.29 (1.05–1.59) and there is heterogeneity in meningioma incidence between individual South Asian ethnicities. Blacks have a higher incidence of pituitary tumours relative to Whites (p<0.01) with an incidence rate ratio of 2.95 (2.37–3.67). There is heterogeneity in pituitary tumour incidence between individual South Asian ethnicities.

**Conclusions:**

We present incidence data of individual tumour types for seven ethnic groups. Current understanding of the aetiology of these tumours cannot explain our results. These findings suggest avenues for further work.

## Introduction

Nervous system and intracranial tumours are a varied group of neoplasms composed of multiple morphological subtypes[1] with very different patterns of behaviour. They represent a significant disease burden in the United Kingdom and globally. There were over 9,000 new cases of nervous system and intracranial tumours in the UK in 2010[2]. Worldwide, it is estimated that there are in excess of 256,000 new cases annually[3].

Relatively little is understood about the aetiology of these tumours. A number of genetic conditions have been identified which predispose patients to developing nervous system and intracranial tumours[4], but research has thus far elucidated few environmental factors. Therefore, it is important to improve our understanding in order to develop more effective preventative strategies and therapies.

Global data reveal wide variation in the incidence of nervous system tumours worldwide, with reported age-standardized rates for different nations varying between 0.0 and 12.7 (men) and 0.0 and 10.7 (women) per 100,000 people[3]. In general, the lowest rates are in Africa and the highest rates are in northern Europe[3]. International comparisons are of limited value due to differences in diagnosis, reporting and registration in different countries. Migrant studies can overcome these limitations as similar diagnostic, reporting and registration procedures are used [5].

Current data regarding ethnic variation in nervous system and intracranial tumour incidence is of reduced utility due to its classification of heterogeneous ethnicities together into broad groups such as White, Asian and Black[6]. This approach does not have the ability to determine associations between individual ethnicities (who have diverse social, cultural and genetic characteristics) and tumour incidence. Data from England report that Blacks and Asians have a significantly lower incidence than Whites, but these data are presented only as a summary of all brain and CNS cancers with no ability to view associations between ethnicity and a specific tumour type[6], reducing their utility.

In order to address the limitations of current work, our objective was to determine the incidence of common nervous system and intracranial tumours for the seven major ethnicities in England (White, British Indian, British Pakistani, British Bangladeshi, Black African, Black Caribbean and Chinese). England is an ideal setting to conduct a study of this type due to its universally accessible healthcare system, high-quality cancer registry and diverse ethnic makeup, with non-White ethnic groups comprising approximately 14% of the population[7].

## Materials and Methods

### Study Design

We used a descriptive epidemiological study design.

### Data collection

The National Cancer Intelligence Network (NCIN) provided data for all cancer registrations from January 2001 to December 2007 for residents of England. For each registration, the following information was given: cancer site coded to the International Classifications of Diseases, 10th Revision (ICD-10)[8], morphology coded to the International Classifications of Diseases of Oncology, 2nd and 3rd Revisions (ICD-O-2 and ICD-O-3)[9, 10], deprivation assessed from the income domain of the Index of Multiple Deprivation 2007 (IMD 2007)[11], age at diagnosis of cancer; sex and ethnicity. We used the mid-year population estimates produced by the Office for National Statistics (ONS) from 2001–2007, stratified by age, sex and ethnicity. Population data stratified by national quintiles of the income domain were provided by ONS based on the 2001 census and the same distributions applied to population data by age, sex and ethnicity for the 2001–2007 mid-year population estimates.

### Classification of ethnicity

NCIN obtained the self-assigned ethnicity for each cancer registration by record linkage to the Hospital Episode Statistics (HES) database. If a cancer registration could not be linked or if ethnicity was missing on the HES database, then ethnicity was assigned using the cancer registry data. Prior to April 2001, ethnicity was classified by HES and the cancer registries according to the codes used in the 1991 census. After April 2001, the codes were amended to those used in the 2001 census, although 1991 ethnicity codes were accepted until 2003. For the analyses presented in this paper, ethnicity was classified as White (White from the 1991 Census and White British from the 2001 Census), Indian, Pakistani, Bangladeshi (with the three groups combined to form the category of ‘South Asian’), Black African, Black Caribbean (again both combined to form the category ‘Black’) and Chinese.

### Classification of tumours

We identified nervous system and intra-cranial tumours as those with ICD-10 codes C70-C72, C75.1-C75.3, D32, D33, D35.2-D35.4, D42, D43 and D44.3-D44.5. We then grouped tumours by site and morphology, converting ICD-O codes from the second to the third edition as necessary. Tumours were grouped into gliomas (ICD-O-3 codes 9380–9481); meningiomas (ICD-O-3 codes 9530–9539); pituitary tumours (ICD-O-3 codes 8140/0, 8202/0, 8260/0, 8270/0, 8271/0, 8272/0, 8280/0, 8281/, 8290/0 and 8300/0) and cranial and paraspinal nerve tumours (ICD-O-3 codes 9560/0, 9540/0, 9540/3, 9571/0, 9571/3).

### Statistical analyses

We estimated age standardized rates (ASRs) of cancer per 100,000 person-years for all ethnic groups using direct standardization to the 1960 Segi world population[12], with age at diagnosis of cancer being classified into six categories: <40, 40–49, 50–59, 60–69, 70–79 and ≥ 80 years. We used Poisson regression to estimate incidence rate ratios (RRs) comparing each ethnic group (and the two combined groups, South Asians and Blacks) to Whites, adjusting for the potential confounders sex, age and income.

When comparing South Asians and Blacks to Whites, we present results as RRs and 99% confidence intervals (CIs). When comparing the individual ethnic groups, results are presented as RRs and 99% floating confidence intervals (FCIs). FCIs were calculated using the method of floating absolute risks[13, 14] and enable valid comparisons between any two ethnic groups, even if neither one is the baseline. We calculated 99% CIs because of multiple tests performed across ethnic groups.

We performed pre-specified subgroup analyses of gliomas. This included subdividing gliomas by sex and tumour type. For the latter, we subdivided gliomas into glioblastomas (ICD-O-3 codes 9440–9442) and other gliomas (ICD-O-3 codes 9381, 9384, 9400, 9401, 9410, 9411, 9420, 9421, 9425). This subdivision was chosen because glioblastoma is the most aggressive form of glioma and it is therefore clinically useful to see data for this as a subgroup.

Tests of heterogeneity of RRs between ethnicities, either overall or restricted to South Asians or Blacks, were performed using likelihood Χ^2^ ratio tests. Tests of heterogeneity of RRs between pre-specified subgroups were performed for South Asians, Blacks and Chinese using a Χ^2^ contrast test. Due to missing ethnicity data for some registered cancers, we completed a sensitivity analysis using multiple imputations of the missing ethnicity data based on income, sex, age and cancer site.

We performed all analyses using Stata V.12 and R statistical software packages

### Graphical presentation of results

Where results are presented in the form of plots, we represent RRs for each ethnic group by squares and their corresponding 99% FCIs by straight lines. For the combined South Asian and Black groups, we show RRs as open diamonds, whose horizontal extent indicates the 99% CI. We placed dashed vertical lines at the value of the RRs for South Asians and Blacks.

### Comparison to rates in countries of origin

We also compared the ASRs for each ethnic group in England to rates from their country or region of origin using data from the Globocan database and from population-based registries within IARC’s Cancer Incidence in Five Continents (CI5), where available[15, 16]. From language data contained in the UK census[17] we can infer that the majority of British Indians are from Gujarat and Punjab, neither of which have population-based cancer registries, so we used figures from Mumbai[18]. Most British Pakistanis are from Kashmir and Punjab[19], but the South Karachi Cancer Registry is the only population-based registry in Pakistan. From language data in the census[17] we also determined that most British Bangladeshis are from Sylhet but there are no population-based cancer registries in Bangladesh. For Blacks, we used Globocan estimates for Sub-Saharan Africa and the Caribbean; there are no population based cancer registries in the main countries of origin. For Chinese, we used the Hong Kong cancer registry.

For these comparisons, in keeping with Globocan and CI5, we examined cancers of the nervous system which are those cancers with ICD codes C70-72. The ASRs report by Globocan and CI5 are also standardized to the Segi world population.

### Ethical Approval

This study was approved by the Oxford Research Ethics Committee (this was a requirement for the data to be released by NCIN). Consent was not obtained because the data were analysed anonymously.

## Results

Demographic data for the study population are presented in Table 1. The mean ages of the non-White ethnic groups were younger than the White population. The non-White ethnic groups were also more deprived than the White population, with the exception of Chinese. British Pakistanis and Bangladeshis were particularly deprived. Nearly all of the White population were born in the UK, as were approximately half of the British Indian, Pakistani, Bangladeshi and Black Caribbean populations. Fewer Chinese and Black Africans were born in the UK.

**Table 1:** Demographic information for the study population, taken from the United Kingdom 2001 census. Low, Middle and High deprivation are the 1st, 2nd-4th and 5th quintile of the income domain of IMD 2007, respectively.

		White		Indian		Pakistani		Bangladeshi		Black African		Black Caribbean		Chinese	
		N	(%)	N	(%)	N	(%)	N	(%)	N	(%)	N	(%)	N	(%)
**Census data for 2001**															
**Total population**		42 747 136	(100.0)	1 028 546	(100.0)	706 539	(100.0)	275 394	(100.0)	475 938	(100.0)	561 246	(100.0)	220 681	(100.0)
**Sex**	Male	20 828 644	(48.7)	511 204	(49.7)	358 043	(50.7)	138 972	(50.5)	229 103	(48.1)	259 881	(46.3)	105 913	(48.0)
**Age**	<45	24 920 845	(58.3)	751 879	(73.1)	590 934	(83.6)	238 801	(86.7)	2 594 104	(72.1)	394 532	(70.3)	168 213	(76.2)
	45–64	10 535 612	(24.7)	208 503	(20.3)	86 169	(12.2)	27 675	(10.1)	665 156	(18.5)	107 250	(19.1)	41 158	(18.7)
	65+	7 290 679	(17.1)	68 164	(6.6)	29 436	(4.2)	8 918	(3.2)	339 754	(9.4)	59 464	(10.6)	11 310	(5.1)
**Deprivation**	Low	7 305 527	(17.1)	347 098	(33.7)	455 710	(64.5)	198 884	(72.2)	277 858	(58.4)	292 537	(52.1)	49 427	(22.4)
	Middle	26 315 786	(61.6)	563 939	(54.8)	222 038	(31.4)	69 325	(25.2)	177 234	(37.2)	245 103	(43.7)	123 994	(56.2)
	High	9 125 823	(21.3)	117 509	(11.4)	28 791	(4.1)	7 185	(2.6)	20 846	(4.4)	23 606	(4.2)	47 260	(21.4)
**Country of Birth**	United Kingdom	41 911 150	(98.0)	472 545	(45.9)	387 198	(54.8)	127 902	(46.4)	161 050	(33.8)	324 764	(57.9)	62 209	(28.2)
	Other	835 986	(2.0)	556 001	(54.1)	319 341	(45.2)	147 492	(53.6)	314 888	(66.2)	236 482	(42.1)	158 472	(71.8)

Table 2 shows the number of tumour cases in England 2001–2007 by tumour type and ethnicity. We identified 42,207 gliomas, meningiomas, pituitary tumours, and cranial and paraspinal nerve tumours in total. Of these, 6,544 cases (15.5%) had no ethnicity recorded and were therefore excluded from our analysis.

**Table 2:** Number of tumour cases in England 2001–2007 by ethnicity, and number of patients with missing ethnicity.

	White		Indian		Pakistani		Bangladeshi		Black African		Black Caribbean		Chinese		All other ethnic groups		No ethnicity recorded		Total
Glioblastomas	10,077	(78.3)	99	(0.8)	59	(0.5)	26	(0.2)	26	(0.2)	58	(0.5)	15	(0.1)	707	(5.5)	1,803	(14.0)	12,870
Other Gliomas	8,893	(73.1)	148	(1.2)	105	(0.9)	27	(0.2)	62	(0.5)	83	(0.7)	39	(0.3)	851	(7.0)	1,950	(16.0)	12,158
Meninges	7,358	(73.5)	94	(0.9)	79	(0.8)	11	(0.1)	58	(0.6)	104	(1.0)	30	(0.3)	672	(6.7)	1,601	(16.0)	10,007
Cranial & Paraspinal nerve	2,317	(72.0)	35	(1.1)	29	(0.9)	8	(0.2)	12	(0.4)	15	(0.5)	13	(0.4)	165	(5.1)	622	(19.3)	3,216
Pituitary	2,795	(70.7)	73	(1.8)	43	(1.1)	2	(0.1)	52	(1.3)	107	(2.7)	22	(0.6)	294	(7.4)	568	(14.4)	3,956
All five cancers	31,440	(74.5)	449	(1.1)	315	(0.7)	74	(0.2)	210	(0.5)	367	(0.9)	119	(0.3)	2,689	(6.4)	6,544	(15.5)	42,207

Footnotes:

Percentages in brackets.

### Incidence by Ethnic Group

**Fig 1:**
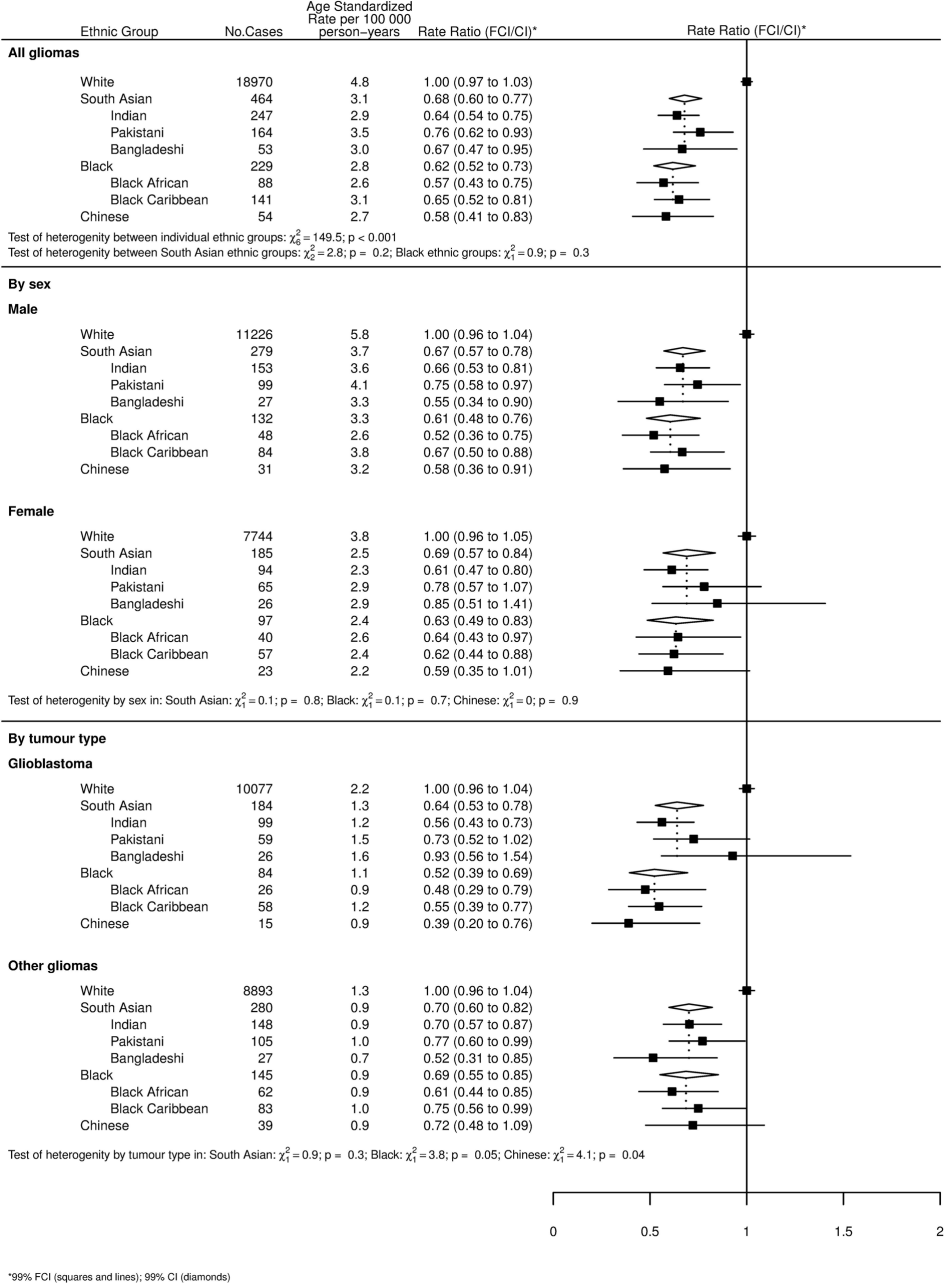
Age-standardized incidence rates and incidence rate ratios by ethnicity for all gliomas, all gliomas by sex, glioblastomas and other gliomas. Tests of heterogeneity by sex, between all ethnicities (White, Indian, Pakistani, Bangladeshi, Black African, Black Caribbean and Chinese) and between the individual Black and South Asian ethnic groups are also shown.

**Fig 2:**
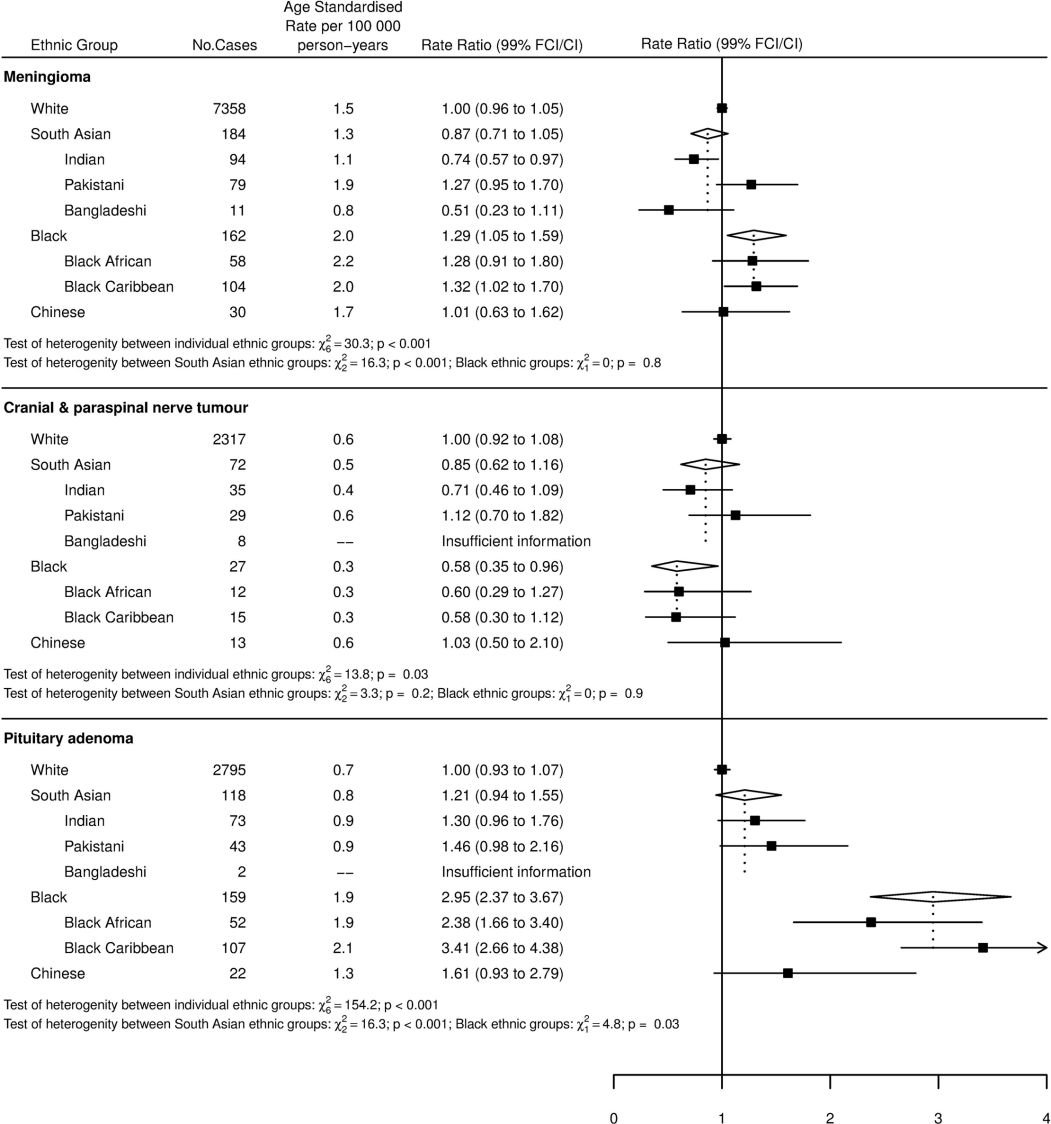
Age-standardized incidence rates and incidence rate ratios for meningioma, cranial and paraspinal nerve tumours and pituitary tumours by ethnicity. Tests of heterogeneity between all ethnicities (White, Indian, Pakistani, Bangladeshi, Black African, Black Caribbean and Chinese) and between the individual Black and South Asian ethnic groups are also shown.

Figs 1 and 2 show age-standardized rates (ASR) and incidence rate ratios (RR) for gliomas and their subgroup, meningiomas, cranial and paraspinal nerve tumours, and pituitary tumours by ethnicity. With reference to the overall tests of heterogeneity between individual ethnic groups for each tumour type, there were significant differences between all individual ethnicities (White, Indian, Pakistani, Bangladeshi, Black African, Black Caribbean and Chinese) for all tumour types (p<0.001 for all tumour types except cranial and paraspinal nerve tumours, where p = 0.03).

Fig 1 shows gliomas, subdivided by sex and tumour type (glioblastomas and other gliomas). Whites had a significantly higher incidence rate of all gliomas than every other ethnicity. There were no differences in rates of glioma between non-White ethnic groups. There was no heterogeneity between the different Black and South Asian ethnic groups. Analysis of all gliomas by sex did not reveal heterogeneity by sex for any of the ethnic groups.

Whites had the highest incidence rate of glioblastoma with South Asians, Blacks and Chinese all having a significantly lower rate. The subgroup analysis of other gliomas (all gliomas, excluding glioblastomas) showed that Whites had a higher rate than all other ethnic groups with the exception of Pakistanis, Black Caribbeans and Chinese. There were no differences in rates between non-White ethnic groups.

The results for meningiomas in Fig 2 show that Blacks had the highest incidence rate, which was significantly higher than the rate for Whites and South Asians. There was no difference in rates between Whites, South Asians and Chinese. Heterogeneity testing between the South Asian ethnic groups revealed significant heterogeneity (p<0.001), with Pakistanis (RR = 1.27) experiencing over double the incidence of tumours compared to Bangladeshis (RR = 0.51). There was no heterogeneity between different Black ethnic groups.

The incidence of cranial and paraspinal nerve tumours by ethnicity, shown in Fig 2, revealed observed differences in incidence rates between ethnicities which were not significant when comparing any two individual ethnicities, but the level of overall variation was significant (p = 0.03).

Pituitary tumours are addressed in Fig 2. This shows that Blacks had a significantly higher incidence rate than Whites and were nearly three times more likely to develop these tumours. Blacks were also more than twice as likely as South Asians to develop pituitary tumours. There were insufficient cases to include Bangladeshis. However, it should be noted that the presence of only two cases for this ethnic group is noticeably lower than for British Indians (RR = 1.30) or British Pakistanis (RR = 1.46), reflected by the finding of significant heterogeneity between South Asian ethnic groups (p<0.001).

### Sensitivity Analysis

We assigned missing ethnicity values by multiple imputation. The results were extremely similar to those presented in Figs 1 and 2. The sensitivity analysis is presented in S1 Fig.

### Comparison to Rates in Countries of Origin

Table 3 shows age-standardized incidence rates of nervous system tumours (ICD-10 codes C70-72) for individual ethnicities in England compared with the incidence rate in the country of origin. Amongst men, all ethnic groups had a higher incidence rate than their country of origin with the exception of Chinese men, where a higher incidence rate in Hong Kong was observed by Globocan. British Bangladeshi and Black African men had a particularly high incidence rate in England compared with their country of origin. Amongst women, most ethnic groups also had a higher incidence rate in England, with the exception of British Indians, where a higher incidence rate was observed in India by Cancer Incidence in Five Continents; Black Caribbeans, where a higher incidence rate was observed in the Caribbean by Globocan; and Chinese, where a higher incidence rate was observed in Hong Kong by Globocan. British Bangladeshi and Black African women had a particularly high incidence rate in England compared to their country of origin.

**Table 3:** Age-standardised incidence rates for nervous system cancers in England compared with incidence rates in country of origin.

Ethnic Group	Male				Female			
	cases	ASR			cases	ASR		
		per 100 000 person years				per 100 000 person years		
		England	Globocan	CIV		England	Globocan	CIV
White	12621	6.3			9047	4.2		
Indian	165	3.9	2.1	3.7	107	2.6	1.2	2.8
Pakistani	105	4.4	3.4	3.3	73	3.2	2.1	2.7
Bangladeshi	32	3.8	1.2		28	3.2	0.7	
Black African	53	3.0	0.9		50	3.2	0.7	
Black Caribbean	99	4.5	3.3		63	2.7	3.3	
Chinese	33	3.5	4.2	3.4	28	2.7	3.7	2.2

Footnotes:

Nervous system cancers defined as ICD-10 codes C70-72.

Incidence rates in country of origin determined by Globocan and Cancer Incidence in Five Continents (CIV).

## Discussion

The objective of this study was to determine the incidence of the most common nervous system and intracranial tumours for the seven major individual ethnicities in England (White, British Indian, British Pakistani, British Bangladeshi, Black African, Black Caribbean and Chinese). This is the first time that data on the five individual ethnic groups which compose the broader South Asian and Black groups in England have been presented.

There are significant differences in incidence between ethnicities for all tumour types. Specifically, there is significant heterogeneity between the South Asian ethnic groups (Indian, Pakistani, Bangladeshi) for both meningiomas and pituitary tumours, which is a novel finding. In addition, we show that Blacks are nearly three times more likely to develop pituitary tumours than Whites, and Whites are significantly more likely to develop gliomas than any other ethnic group, and are also more likely to develop glioblastoma, the commonest and most aggressive form of adult primary brain tumour. Blacks are significantly more likely to develop meningioma than Whites or South Asians.

We report that Whites are significantly more likely to develop gliomas than South Asians, Blacks or Chinese, all of whom have a similar incidence rate. This finding is broadly consistent with published data from the USA[20]. Glioma results from multifactorial inheritance, with both environmental and genetic factors at work[21], and there are few proven risk factors[22]. The role of genetic mechanisms in glioma development is still being understood, and the relationship between ethnicity and genetic risk factors is not yet clear. An increased incidence of glioma has been observed in individuals with the monogenic Mendelian disorders Neurofibromatosis 1 &2, Tuberous Sclerosis, Lynch Syndrome, Li-Fraumeni Syndrome, Melanoma-neural system tumour syndrome and Ollier disease[23, 24]. However, we could not find evidence that the incidence of these conditions varies by ethnicity [25–27], and these conditions represent only a small percentage of glioma incidence overall[23]. Genome-wide association studies have identified eight single nucleotide polymorphisms (SNPs) in seven genes which are significantly associated with glioma development[24]. Of these, it has been reported that rs2736100-C occurs more frequently in Caucasian than Asian populations[28, 29], which might partly contribute to the higher incidence of glioma in Whites observed here, and another concluded that the SNP rs6010620 might account for an IRR of 1.34 for Whites compared to East Asians for glioma[30]. Other work has indicated that gliomas from non-White patients contained mutations in the pP53 tumour suppressor gene more frequently than those from White patients[31]. Polymorphisms in the ERCC1 gene have been shown to influence glioma susceptibility[32] and it has previously been demonstrated that the frequency of these polymorphisms varies by ethnicity[33]. Work indicates that the Arg399Gln polymorphism in the XRCC1 DNA repair gene may contribute to the likelihood of developing glioma in Asian patients but not in Caucasians[34]. Ethnic variation in other possible genetic risk factors has been reported. For example, it has been suggested that individuals with the B*07 and B*07-Cw*07 haplotypes, which occur most commonly in Caucasians, have a higher incidence of glioblastoma multiforme and have a worse prognosis [35]. Additionally, work has suggested that homozygous deletion of the p16/MST-1/CDKN2 tumour suppressor gene is less common in Japanese patients with glioma than among Caucasian patients[36]. With reference to environmental factors, exposure to ionising radiation is a known risk factor for glioma[22, 24] and individuals who are irradiated at a younger age are also at increased risk[37]. This might explain a small part of our results because evidence suggests that ethnic groups are less able to access UK healthcare[38]. Therefore, it is possible that ethnic minorities are less likely to receive ionising radiation, resulting in a lower incidence rate of glioma. Additionally, a larger number of ethnic minority than White individuals are born outside the UK[39] and a significant proportion of people from ethnic minorities travel from countries with poorer healthcare access than the UK. Equally, this factor might partly explain our results through detection bias if less gliomas are diagnosed in non-White individuals due to reduced access to healthcare. Previous work suggests that individuals with a non-CNS primary tumour in childhood are 6.5 times more likely to develop glioma in later life[40], and survivors of CNS primary tumours in childhood are twelve times more likely to develop subsequent gliomas[40]. This may be due to radiotherapy or chemotherapy received for the childhood tumour[37]. Data from the USA suggest that Blacks and Asian/Pacific Islanders have a lower incidence rate of childhood cancer than Whites[41], and international data suggest that incidence rates of childhood cancer in many Asian and African nations are lower than in the UK[42], although this is likely to be unreliable due to poorer diagnosis and reporting. Nevertheless, the seemingly lower incidence of childhood cancer among non-White ethnic groups might contribute to the results we present here, although the effect is likely to be very small. Atopic disease is protective against glioma[24, 43], possibly due to increased TH2 cytokine production which may protect against glioma development via mediation of the immune response[43]. South Asians and Blacks have been shown to have higher rates of new asthma consultations than Whites[44]. This may reflect a lower incidence of asthma in Whites meaning that they lack the protection conferred by atopy and therefore have higher incidence of glioma. Initial work suggests that HRT use by women might be associated with lower glioma incidence[45]. Studies indicate that White women are more likely to use HRT[46], which cannot explain Whites’ higher incidence of glioma. Additionally, there is weak evidence to suggest that old age at menarche is associated with increased risk of glioma[45, 47]. On average, White women are older at menarche[48], possibly providing a partial explanation for our results. A meta-analysis has demonstrated that children with a birth weight of over 4kg have 38% increased risk of developing astrocytoma, a form of glioma[49]. In the UK, South Asian babies are 2.5 times more likely to be low birth weight than White babies, and Black babies are 60% more likely to be low birth weight than White babies[50]. This could provide a partial explanation for our results. The significantly lower incidence rate of glioma in Chinese compared to Whites has been reflected by other studies. For example, the incidence of malignant glioma among Hong Kong residents of Chinese origin has previously been shown to be 1/100,000 people[51], and the incidence of glioma in wider China is between 1-4/100,000 people[52]. This compares to an incidence of glioma between 4.67 and 5.73/100,000 people in Europe[53–55]. There is no clear evidence to explain why the incidence of glioma is significantly lower in Chinese. As noted above, the relationship between ethnicity and genetic risk factors is not yet clear, but there are suggestions that variations in SNPs might partly account for the reduced incidence observed in Chinese[28–30]. With reference to environmental factors and the protective effect of atopic disease[24], there are no data regarding the prevalence of atopic disease in Chinese living in the UK. However, China has been shown to have a low reported prevalence of symptoms of atopic eczema [56] and asthma [57] in children when compared to other countries. Atopic disease has a mixed environmental and genetic pathogenesis, therefore the lower observed prevalence of atopic disease in China suggests that Chinese patients in the UK may be genetically less likely to develop atopic disease. This, therefore, does not explain our results. With regard to ionizing radiation and increased risk of glioma, the evidence discussed earlier that ethnic minorities have poorer access to healthcare in the UK[38] might also apply here.

We found significant differences in the incidence rate of meningioma between ethnic groups, with Blacks having the highest incidence rate. Additionally, there is significant heterogeneity in incidence rate between the individual South Asian ethnic groups, with Pakistanis having the highest incidence rate. Published studies report that Blacks have the highest incidence rate of meningioma[20], consistent with our findings. Genetic and environmental risk factors have been identified for meningioma, some of which might partly explain our results. People with Turner syndrome[58] and Neurofibromatoses[59] have a higher incidence of meningioma. However, there is no evidence of variation in incidence of these conditionsby ethnicity[26, 60]. Analysis of data from the Interphone study identified twelve SNPs associated with development of meningioma[61], with a later meta-analysis revealing that Caucasians have a significantly higher risk of meningioma if they carry the CT genotype of the Methylenetetrahydrofolate reductase gene, and are more likely to carry this genotype than controls[62]. However, the overall relationship between ethnicity and genetic risk factors is not yet clear. With reference to environmental factors, ionising radiation is linked to meningioma development[37, 59]. This is unlikely to contribute to our findings as evidence suggests that ethnic minorities have poorer access healthcare in the UK[38]. Previous work indicates that meningioma risk is increased in individuals who have survived childhood tumours[37]. As discussed above, non-White ethnicities have a lower incidence of childhood tumours, which cannot explain our results[41, 42]. Meningioma risk might be lower in those with a history of allergy[43], although this association may be limited to eczema alone[63]. There is no clear evidence of ethnic variation in eczema incidence, although there is a suggestion that Black Caribbeans may be at increased risk[64], which is not what we might expect from our results. Initial work indicates that meningioma risk might be higher in those who have used hormone replacement therapy (HRT)[65]. There is some evidence that White women are more likely to use HRT[46], which does not explain our results. Multiparity may also be a risk factor for meningioma[65]. In the UK, Pakistani and Bangladeshi women are likely to have the highest fertility rate[66]. We report that Bangladeshis have a particularly high rate and Pakistanis a particularly low rate of meningioma, which cannot be explained by ethnic variation in fertility rate. Obesity might increase women’s risk of developing meningioma[67]. Depending on the measure used, evidence suggests that Black African or Bangladeshi women have the highest prevalence of obesity[68]. This could explain why we report that Black Africans have the highest rate of meningioma, but not our result that Bangladeshis have the lowest.

We present results which show overall ethnic heterogeneity in incidence of cranial and paraspinal nerve tumours. There were no significant differences when comparing any two individual ethnic groups directly. There were insufficient data to include Bangladeshis. Most individuals with Neurofibromatosis Type 2 (NF2) develop acoustic neuromas[26], but there is no evidence to show that incidence varies by ethnicity[26]. SNPs have also been identified which are associated with both increased and reduced risk of acoustic neuroma[69]. However, the relationship between genetic risk and ethnicity is still being understood. Recent work hints that allergy may be protective against development of acoustic neuroma[70]. As noted earlier, Blacks and South Asians have higher rates of new consultations for asthma than Whites[44], which could partly explain our results, with the exception of Pakistanis. There is evidence to suggest that smoking is protective against acoustic neuroma[71]. ONS data indicate that South Asians, Blacks and Chinese have a similar prevalence of smoking, which is lower than Whites[72] and therefore does not provide a clear explanation for our results.

For pituitary tumours, there is significant heterogeneity between the individual South Asian ethnic groups, with only two cases in the Bangladeshi population, far fewer than in Indians or Pakistanis. This is a new finding which requires further investigation. Blacks have the highest incidence rate of pituitary tumours. This finding is reflected by other published data[20, 73]. The higher incidence rate of pituitary tumours amongst Blacks may be genetic as opposed to environmental, because it has been reported that African Blacks as well as American Blacks have a higher incidence rate than American Whites[73]. This theory is supported by the current literature which report no known environmental risk factors for pituitary tumours[74]. Several genetic syndromes have been identified which increase the risk of pituitary tumours, including Multiple Endocrine Neoplasia Type 1 (MEN1), McCune-Albright syndrome and Carney complex, but these conditions have no ethnic predilection [75–77]. A number of other genetic mechanisms are implicated in the development of pituitary tumours[78], but the relationship of these mechanisms to ethnicity is currently unclear.

A strength of this study is our use of accurate ethnicity data. These data are self-reported by patients and obtained via linkage to the HES database. This is an advance compared with determination of ethnicity via birth certificates or name analysis. Birth certificates are unreliable due to the assumption that everyone born in a particular location is of a certain ethnicity, and because a significant proportion of non-white people are born within the UK[79]. Name analysis is unreliable due to the inability to distinguish between specific ethnic groups within the broad categories of South Asian, Chinese and Black on the basis of name alone. Our study also benefits from use of the ICD-O-3 classification of tumours which is more specific than the ICD-10 classification. This allows us to draw conclusions with greater scientific value. This is in contrast to previous British studies relating to ethnicity and CNS cancer which use the ICD-10 classification[6]. ICD-10 is based on location without reference to histology and hence is less accurate. To our knowledge, this is the first study which presents data on nervous system and intracranial tumours using this classification system and ethnic categorization.

Limitations of this paper include being unable to assign ethnicity to 15.5% of individuals. However, this is lower than previous studies[6, 80] and the sensitivity analysis demonstrated that this did not change our results. Over the last two decades the quality of ethnicity data in HES has improved significantly, illustrated by a reduction in the percentage of missing ethnicity values from 35% to less than 10% between 1998 and 2009. Evaluation also shows that no ethnicity is significantly misrepresented in English HES data [81, 82]. In addition, a lack of data on exposure of individuals to risk factors made explanation of our results more challenging. Despite this, we were able to draw broad conclusions by examining population-level exposure to risk factors. It is difficult to draw reliable comparisons between incidence rates in non-White ethnic groups in England and rates in the country of origin. This is due to under-diagnosis and reporting of malignancies in a number of these countries due to poorer access to healthcare and lower quality cancer registries. Rural-urban status of individuals may represent a meaningful confounder. However, it was not possible to adjust for this as the Office for National Statistics do not provide these data. This is therefore a limitation of our approach. Finally, it should be noted that eight years of data are too few to investigate possible cohort effects on tumour incidence.

Our findings may help to identify new environmental risk factors for these tumours. For ethnic groups with a particularly high incidence of a certain tumour, it may be possible to examine environmental factors which are more common in this ethnic group. It is important to note that this study contains only English patients, and therefore the generalizability of these findings might be limited outside of England. We demonstrate considerable variation in incidence rates of nervous system and intracranial tumours by ethnicity. However, current knowledge of the aetiology and risk factors for these tumours is not sufficient to explain our findings. Therefore, there is considerable scope for further exploration in order to optimise prevention and diagnosis of these tumours in future.

## Supporting Information

S1 Fig Sensitivity analysis demonstrating age-standardized incidence rates and incidence rate ratios for glioma, meningioma, cranial and paraspinal nerve and pituitary tumours by ethnicity. (TIF)
