# Supplementary figures and images for: Nervous System and Intracranial Tumour Incidence by Ethnicity in England, 2001–2007: A Descriptive Epidemiological Study
Source: PLoS One. 2016 May 2;11(5):e0154347. doi: 10.1371/journal.pone.0154347 (PMC4852951; doi:10.1371/journal.pone.0154347)

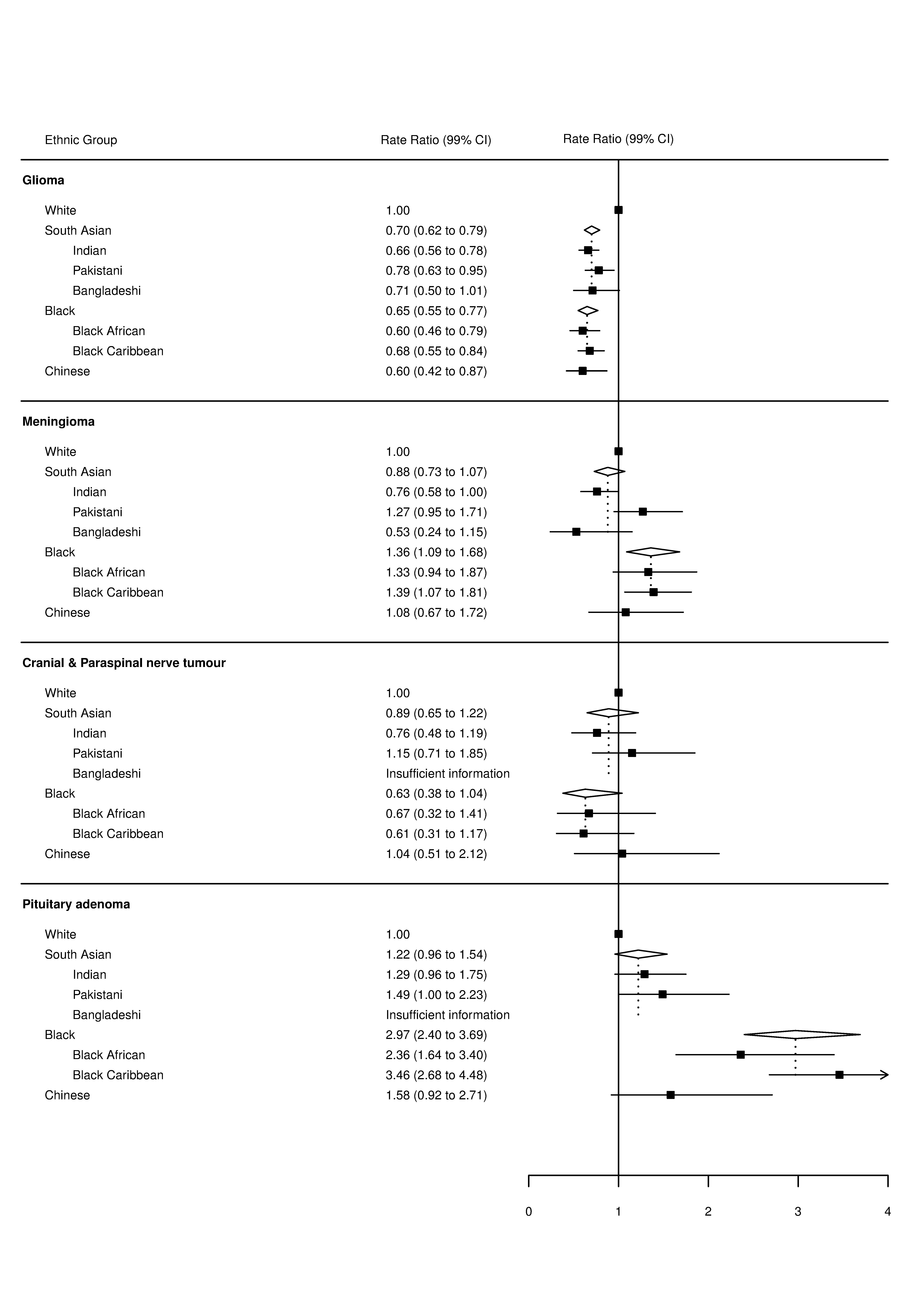

Supplement: S1 Fig — (TIF) [file pone.0154347.s001.tif]
